# The effect of non-organophosphate household pesticides exposure during pregnancy on infants birth sizes and growth rate: a cohort study

**DOI:** 10.1186/s12884-020-03162-w

**Published:** 2020-08-20

**Authors:** Frida Soesanti, Nikmah S. Idris, Kerstin Klipstein-Grobusch, Aryono Hendarto, Diederick E. Grobbee, Cuno S. P. M. Uiterwaal

**Affiliations:** 1 Department of Child Health, Faculty of Medicine, Universitas Indonesia/Cipto Mangunkusumo General Hospital, Jakarta, Indonesia; 2 Julius Global Health, Julius Center for Health Sciences and Primary Care, University Medical Center, Utrecht University, Utrecht, The Netherlands; 3 Division of Epidemiology and Biostatistics, School of Public Health, Faculty of Health Sciences, University of the Witwatersrand, Johannesburg, South Africa

## Abstract

**Background:**

To date, there is limited evidence on the effect of antenatal exposure to non-organophosphate household pesticides on infant health. Our hypothesis is that antenatal exposure to non-organophosphate household pesticides will be associated with birth sizes and infant growth rate.

**Methods:**

In this prospective cohort study, 284 mother-infant pairs were studied. Mothers were recruited at the third trimester in two primary care centers and one private hospital in Jakarta, Indonesia. Mothers filled out questionnaires about exposure to non-organophosphate household pesticides at the 3rd trimester of pregnancy. Birth weight and length were measured at birth. Afterwards, the weight, height, and head circumference (HC) were measured at 7 days, 1, 2, 4, and 6 months of age. Linear mixed modeling and linear regression was performed to calculate growth rate of each infant. Multivariable linear regression adjusted for confounders was used to assess the association between household pesticides exposure and birth sizes and infant growth rate.

**Results:**

Based on self-report questionnaires, 133 (46.8%) mothers were exposed to household pesticides during pregnancy. The mean HC at day 7 in the exposed group was − 7.1 mm (95%CI -13.1;-1.2) lower than in the non-exposed group. The difference was more prominent in the non-mosquito pesticide group (linear regression coefficient: − 22.1 mm, 95%CI -36.5;-7.6). No material associations were found between antenatal exposure to household pesticides with other growth measures, including weight gain, length gain, HC increment and weight-to-length gain rates. No modification of effects by breastfeeding was found.

**Conclusions:**

Our findings suggest that antenatal exposure to household non-organophosphate pesticides is associated with smaller head circumference at birth.

## Background

Endocrine disrupting chemicals (EDC) is the term used to describe substances that can potentially interfere with the endocrine systems [1, 2]. EDCs are divided into major classes of chemicals including various types of pesticides, industrial chemicals, plastic packaging components, fuels and other materials that are used in daily life [2]. The exposures may occur early in life since pregnant woman may transfer EDCs through the placenta to the fetus [1, 3, 4]. EDCs may have trans-generational effects with impacts on health mediated through epigenetic mechanisms [1–3].

While there is substantial evidence on animal and human studies regarding the effects of endocrine disrupting organophosphate pesticides to the male reproductive system [5, 6], puberty [7], thyroid [8–10], and obesity [11]; there is little evidence on the health effects of early exposure to household pesticides during pregnancy on infant growth. Prenatal exposure to organophosphate insecticides is associated with neurobehavioral deficits in humans and animal models. Prenatal exposures to organophosphate pesticides have been linked to smaller head size [12], lower birth weight [13], attention problems, neurodevelopmental disorder and a significant reduction in childhood IQ [1, 14, 15].

The use of organophosphate pesticides has been banned for household purposes [16]. Currently, most of household pesticides contain either pyrethroids, propoxur, transfluthrin, or in combinations [16, 17]. These pesticides tend to degrade rapidly in the outdoor environment but half-lives appear to be longer indoors. Prenatal exposure to pyrethroids and propoxur have been linked to neurodevelopmental disorders in early life [17], while in animal studies, chronic toxicity of propoxur has been associated with decreased weight gain [18]. In rats, transfluthrin has been shown to have low acute and dermal toxicity, but no evidence of teratogenicity, fetoxicity or reproductive toxicity [19].

There are no formal data on the use of non-organophosphate household pesticides in Indonesia, but we assume that the use of household pesticides such as coil and spray are high because Indonesia is endemic for dengue and malaria, both are transmitted by mosquitos. Therefore, controlling mosquitos’ population may reduce dengue and malaria infection. Based on the Indonesian Basic Health Survey 2013 (Riskesdas 2013), most of Indonesian households use the coil (48.4%), followed by repellant (16.9%) and spray (12.2%) pesticides [20]. The coil is the first choice among Indonesian households in terms of affordability [20]. The active ingredients of household coil and spray pesticides mainly are pyrethroids and transfluthrin. Crowding and poor ventilation might increase the level of exposure of these degradable pesticides.

This was a preliminary study to explore the association of exposure to non-organophosphate household pesticides in mothers during pregnancy with the birth sizes and growth rate of their infants in the first 6 month of life. Secondarily, our study aimed to assess any association of exposure to non-organophosphate household pesticides during pregnancy with other health consequences in the offspring including respiratory symptoms, skin problems, and the rate of infections.

## Methods

This was a prospective cohort study as part of the Breastfeeding Attitude and Volume Optimization (BRAVO) study (NCT01566812) in Jakarta, Indonesia [21].The participant enrollment was performed from June 2012 to January 2017*.* From 718 participants enrolled in BRAVO study, initially, 282 participants were excluded due to incomplete data on exposure. Afterwards, 152 participants were excluded due to incomplete infant’s growth measurement, thus we included 284 pairs of mother-infant with complete data on exposure and growth measurement of their infants for analysis. Both populations were comparable on important prognostic factors (supplemental data). Pregnant women in three centers, i.e., one private maternal and children hospital (Budi Kemuliaan Hospital) and two primary care centers in Senen and Jatinegara districts, Jakarta, Indonesia were recruited for participation in the BRAVO study during their 3rd trimester antenatal care visit. BRAVO was ethically approved by the Institutional Review Board of the Faculty of Medicine Universitas Indonesia/Cipto Mangunkusumo Hospital, Jakarta, Indonesia (reference number: 913/UN2.F1/ETIK/X/2012) [21].Informed consent was obtained from all study participants prior to study enrolment [21].

Maternal characteristics including maternal age, parity, history of abortion, family income, level of education, weight gain during pregnancy (expressed as ΔBMI: the difference between BMI at labor and BMI prior to pregnancy), and alcohol or illicit drug use in pregnancy were obtained from self-report questionnaires filled out by pregnant women at recruitment [21]. Gestational age at delivery, Apgar score, maternal hypertension and diabetes (pre-existing or gestational), certain neonatal morbidities (special nursery care requirements, including sepsis, respiratory distress, and hyperbilirubinemia) were obtained from medical records [21].

### Measurement of exposure

Exposure to household pesticides was obtained by validated self-report questionnaires filled out by the pregnant women at recruitment [21]. Mothers were considered as exposed to household pesticides when they use mosquito insecticides, or other pesticides to eliminate cockroaches or other insects during their pregnancy. Due to the all year high incidence of dengue in Jakarta, we assume that the use of pesticides is a routine manner. We decided to classify the pesticides exposure into three groups as exposed to mosquito vs non mosquito vs combination of both type considering that e.g. pesticides used against cockroaches might be more potent than pesticides used against mosquitos. No data on the type or brand of household pesticides was recorded [21]. Exposure to other possible EDCs including water sources, fuel usage at home, and garbage burning were recorded [21]. Traveling using open vehicles (motorcycle or bajaj) without using a face mask during travelling was recorded as proxy to exposure to traffic air pollution [21]. Data on active and passive smoking in mothers during pregnancy were also recorded [21].

### Birth sizes and post natal growth measurement

Birth and length were measured at birth, while head circumference (HC) was measured at day 7 after birth. Afterwards, the weight, height, and HC were measured at 7 days, 1, 2, 4, and 6 months of age. All the measurements were performed twice according to standardized procedures and then averaged. Infants with at least two measurements (other than at birth and day 7) available within the first 6 months of life were included in the analyses [22, 23]. Subsequently, linear mixed modeling was performed with extraction of estimated random slopes per child for weight, length, and HC. Linear regression was performed to calculate the predicted values per child, giving the estimated length gain rate, weight gain rate and HC increment per child. Weight gain rate was expressed in grams per day, while the length gain rate and HC increment were expressed in cm per months [22, 23]. Weight gain rate adjusted for length gain rate (WLG), reflecting excess weight gain, was assessed for each child by deriving internal Z-score in our study population and calculating the standardized residuals from the linear regression model with weight gain as the dependent variable and length gain as the independent variable [22, 23].

### Covariates

Socio-economic status (SES) i.e. household income and level of education, mother’s age at pregnancy, BMI increment during pregnancy were considered as covariates in this study. Other explored confounders were active smoking and passive smoking during pregnancy, household water sources, fuel sources, exposure to traffic air pollution, and garbage burning. All of the possible covariates were treated as categorical, excluding mother’s age and BMI increment. Breastfeeding was considered as a potential effect modifier [24]. Information on breastfeeding was obtained by questionnaire at 1, 2, 4 and 6 months of infant’s age.

### Other infant health outcomes

Episode of fever, respiratory symptoms, gastrointrointestinal, skin and eye infection symptoms were collected by use of an infection diary filled by mothers at 1, 2, 4 and 6 months of infant’s age. Fever was subjectively defined as increased temperature that needed antipyretic drug or axilla temperature > 37.5 °C. Gastrointestinal symptoms were recorded as any episodes of vomiting, diarrhea or loose stool. Any cough episodes, runny nose, or dyspnea were recorded as respiratory symptoms. Skin symptoms were recorded as eczema or acute urticaria, while eye symptoms defined as any discharge in one of both eyes or conjunctival injection (redness in the white portions).

### Statistical analysis

Baseline characteristics were tabulated by exposure (yes/no) to household pesticides. Continuous variables were expressed as mean and standard deviation or median and interquartile range if distributions were skewed. Group differences were estimated and tested by independent groups t-test, chi-square test, or Fisher’s exact test where appropriate and *p* values were provided.

Multivariable linear regression adjusted for confounders was used to assess the associations between household pesticides exposure and birth sizes (weight, length, HC) and between household pesticides exposure and growth rate (weight, length, HC, and WLG). To that end, dummy variables were created for categories of household pesticides exposures (mosquito pesticides, non-mosquito pesticides, and combined pesticides groups) and simultaneously entered into the models as independent variables. Possible interaction between household pesticides categories and breastfeeding at 6 months of age on growth rates was tested by adding a product term of (dummy variable of house hold categories * breast feeding at 6 months) to the models. The difference in the incidence of infections in early life (fever, respiratory, gastrointestinal, skin, and eye infection) between exposed and non-exposed to household pesticides group were explored using the chi square test.

Statistical significance was assumed if 95% confidence intervals did not include the estimation null values, corresponding to a two-sided *p* value of < 0.05. Statistical analyses were conducted using IBM SPSS version 24 for Mac.

## Results

Baseline characteristics of the study population are shown in Table 1. One hundred thirty three (46.8%) of 284 mothers were exposed to household pesticides during pregnancy. There were no differences in maternal age, history of abortion, parity, BMI increment during pregnancy and SES between the exposed and non-exposed groups. There were no significant differences in the use of other possible EDCs. However more subjects in the exposed group burned their garbage compared to the unexposed. Infants born from non-exposed group were sligthly heavier, taller and had larger head circumference at birth compare to those of exposed group, eventhough the difference was not significant.

**Table 1:** Baseline subject characteristics

		Use of household pesticides during pregnancy		
Variable		Non-exposed (*n* = 151)	Exposed (*n* = 133)	*p*-value
INFANT				
Male sex, n (%)		86 (58.5)	63 (48.8)	0.11
Birth weight (grams), (range)		3149 (380), (range: 2150–4300)	3070 (432), (range: 2340–4150)	0.11
Birth length (cm), (range)		48.44 (1.68) (range: 43–54)	48.08 (2.12) (range: 45–52)	0.14
Head circumference (cm), (range)		34.38 (1.78), (range 26–39)	33.89 (2.12) (range 23–37)	0.08
Gestational age (weeks), (range)		38.9 (1.5), (range: 35–42)	38.9 (1.3), (range: 36–42)	2.1 0.96
Breastfed until 6 months, n (%)		100 (82.6)	85 (78.7)	0.67
MOTHERS				
Maternal age (years)		28.0 (5.2)	28.8 (6.2)	0.29
Parity^a^		2 (2)	2 (2)	0.24
History of abortion, n (%)		13 (9.4)	21 (17.6)	0.16
BMI mother before pregnancy (kg/m^2^)		26.2 (4.6)	26.3 (5.1)	0.88
BMI increment by pregnancy (kg/m^2^)		3.9 (2.3)	3.6 (2.6)	0.40
Family income (USD/month), n (%)	>223^b^	19 (12.6)	22 (17.1)	0.18
Educational level mother, n (%)	University level Senior high school	12 (8.0) 106 (70.7)	9 (6.8) 82 (62.1)	0.42
Travel using open vehicles, n (%)		138 (91.4)	127 (95.5)	0.31
Travel without face mask, n (%)		88 (58.3)	77 (57.9)	0.59
Passive smoking during pregnancy, n (%)		110 (74.8)	100 (76.3)	0.77
Actively smoking during pregnancy, n (%)		2 (1.3)	3 (2.3)	0.67
Home fuel resources n (%)				
Kerosene		11 (7.3)	12 (9.0)	0.61
Gas		133 (88.1)	118 (88.7)	
No garbage burning, n (%)		134 (88.7)	99 (74.4)	0.02
Water resources, n %				
City pipe		104 (68.9)	95 (71.4)	0.53

Note: numbers are mean (SD) otherwise indicated, ^a^: median (interquartile range), ^b^family income 223 USD/month is the Indonesian minimum monthly wedges per capita

Table 2 shows the association between antenatal exposure to household pesticides with birth sizes. In the final adjustment, the HC at day 7 in the exposed group was significantly lower by 7.1 mm than in the non-exposed group (95% CI of − 13.1;-1.2). Mean birth weight and birth length of infants in the exposed group were lower than those of the non-exposed (106.3 g and 3.7 mm lower, respectively), but these were borderline statistically significant after adjustments. From the subgroup analysis (Table 2), birth weight was lower in the group who were exposed to “mosquito pesticides only” compared to the other groups (adjusted coefficient: − 123.5 g), while the HC was significantly lower in the group that was exposed to non-mosquito pesticides only (adjusted coefficient: − 22.1 mm, 95% CI of − 36.5;-7.6).

**Table 2:** Association between exposure to household pesticides during pregnancy with birth anthropometrics

Exposure	Model	Linear regression coefficients (95% confidence interval)		
		Birth weight (g)	Birth length (mm)	Head circumference (mm)
Non-Exposed (*n* = 151)		Reference	Reference	Reference
Exposed to Household pesticides (*n* = 133)	Crude	− 121.4 (− 227.6; −15.2)^*^	−4.0 (−9.3;1.4)	−6.4 (−12.1;-0.7)^*^
	Model 1	−112.3 (−219.6;-5.1)^*^	−3.7 (−9.1;1.8)	− 6.6 (− 12.3:-0.8)^*^
	Model 2	−106.3 (−215.8; 3.2)	−3.7 (− 9.3;1.9)	−7.1 (− 13.1;-1.2)^*^
Exposed to mosquito insecticides only (*n* = 87)	Crude	− 134.1 (− 255.7;-12.5)^*^	−5.3 (− 11.4;0.7)	−4.0 (− 10.4;2.4)
	Model 1	− 130.2 (− 253.1;-7.2)^*^	−5.2 (− 11.3;0.9)	− 4.3 (− 10.9;2.0)
	Model 2	−123.5 (−248.8;1.7)	− 4.8 (− 11.1;1.1)	−4.7 (− 11.3;2.0)
Exposed to pesticides other than mosquito insecticides (*n* = 15)	Crude	−82.1 (− 332.9;168.7)	−6.5(− 19.1;6.0)	−22.2 (− 36.5;-7.8)^*^
	Model 1	− 57.7 (− 308.4;193.0)	−5.3 (− 17.9;7.3)	− 21.8 (− 36.3;-7.4)^*^
	Model 2	− 57.9 (− 309.1;193.3)	−5.7 (− 18.4;7.0)	−22.1 (− 36.5;-7.6) ^*^
Exposed to both mosquito insecticides and other pesticides (*n* = 31)	Crude	− 72.1 (− 246.9;102.7)	2.4 (6.3;11.1)	− 5.7 (− 14.9;3.5)
	Model 1	−71.4 (− 247.8;105.5)	2.1 (6.7;10.9)	−6.6 (− 15.9;2.8)
	Model 2	−59.8 (− 241.4; 103.2)	1.7 (7.5;10.1)	− 6.7 (− 16.2;3.0)

Model 1: adjusted for SES, mother’s age at pregnancy

Model 2: adjusted for SES, mother’s age at pregnancy, household SHS exposure, water sources, fuel sources, garbage burning, and use of open vehicle

*: *p* < 0.05

Infants of exposed mothers continued to have lower weight gain rate, WLG rate, and HC increment than infants in the non-exposed group, but none of these were statistically significant (adjusted coefficients of − 0.17 g/day, − 0.08 mm/month, − 0.05 z-score/day, respectively). Infants of exposed mothers gained more length than the non-exposed group, but this was not statistically significant (adjusted coefficient: 0.04 mm/month) (Table 3). None of the subgroup analyses showed significant differences between the exposed and non-exposed group in terms of weight gain rate, length gain rate, HC increment and WLG rate. There was no significant interaction between the household pesticide categories and breastfeeding on weight gain, height gain, HC increment and WLG rates (all product term *p*-values were > 0.05).

**Table 3:** Associations between exposure to household pesticides during pregnancy with growth rate in the first 6 months of life

	Model	Linear regression coefficients (95% confidence interval)			
		Weight gain rate (g/day)	Length gain rate (mm/month)	HC increment (mm/month)	WLG (z-score/day)
Non-Exposed (*n* = 151)		Reference	Reference	Reference	Reference
Exposed to house hold pesticides (*n* = 133)	Crude	− 0.13 (− 1.05;0.79)	0.06 (− 0.10;0.22)	− 0.10 (− 0.28;0.07)	− 0.03 (− 0.31;0.25)
	Model 1	− 0.13 (− 1.05;0.79)	0.04 (− 0.12;0.20)	−0.09 (− 0.27;0.09)	−0.05 (− 0.34;0.23)
	Model 2	−0.20 (− 1.17;0.77)	0.03 (− 0.14;0.19)	−0.08 (− 0.27;0.10)	−0.06 (− 0.35;0.24)
Exposed to mosquito insecticides only (*n* = 87)	Crude	−0.03 (− 1.02;0.96)	0.10 (− 0.06;0.27)	−0.12 (− 0.31;0.08)	0.005 (− 0.296;0.306)
	Model 1	− 0.01 (− 1.02;0.99)	0.09 (− 0.08;0.25)	−0.10 (− 0.30;0.10)	0.007 (− 0.298;0.312)
	Model 2	− 0.004 (− 1.04;1.03)	0.07 (− 0.10;0.24)	−0.10 (− 0.30;0.10)	0.007 (− 0.307;0.321)
Exposed to pesticides other than mosquito insecticides (*n* = 15)	Crude	0.39 (−1.59;2.38)	0.02 (− 0.31;0.35)	−0.29 (− 0.68;0.10)	0.12 (− 0.48;0.74)
	Model 1	0.21 (− 1.79;2.21)	− 0.01 (− 0.34;0.32)	−0.28 (− 0.68;0.11)	0.06 (− 0.55;0.67)
	Model 2	0.22 (− 1.81;2.25)	− 0.01 (− 0.35;0.32)	−0.29 (− 0.68;0.11)	0.07 (− 0.55;0.68)
Exposed to both mosquito insecticides and other pesticides (*n* = 31)	Crude	0.03 (−1.39;1.44)	− 0.09 (− 0.32;0.15)	0.12 (− 0.16;0.39)	−0.03 (− 0.43;0.43)
	Model 1	−0.12 (− 1.15;1.31)	−0.12 (− 0.20;0.05)	0.14 (− 0.14;0.42)	−0.05 (− 0.49;0.38)
	Model 2	−0.03(− 1.54;1.47)	−0.17 (− 0.20;0.05)	0.17 (− 0.12;0.47)	−0.03 (− 0.49;0.43)

Model 1: adjusted for SES and mother’s age at pregnancy

Model 2: adjusted for SES, mother’s age at pregnancy, breastfed for 6 months, house hold SHS exposure, water sources, fuel sources, garbage burning, and usage of open vehicle

Infants in the exposed group had more skin problems (defined as eczema or acute urticaria) compared to infants in the non-exposed group (32.5% vs 21.2%, *p* = 0.04) (Table 4). Further analysis showed that there was no difference in the incidence of skin problems in early life between the three categories of exposure (*p*-value of 0.30, 0.52, and 0.10, respectively). No interaction with breastfeeding was found. There were no statistically significant differences in the episodes of fever, respiratory symptoms, gastrointestinal symptoms and eye problems in exposed and non-exposed groups.

**Table 4:** Associations between exposure to household pesticides during pregnancy with episodes of infections in early life

	Use of household pesticides during pregnancy		
Episodes of infections in early life	Non-exposed (*n* = 151)	Exposed (*n* = 133)	*p*-value
Fever, n (%)	77 (50.9)	66 (49.6)	0.97
Respiratory symptoms, n (%)	125 (86.8)	108 (87.8)	0.81
Gastrointestinal symptoms, n (%)	61 (42.1)	59 (47.6)	0.37
Skin problems, n (%)	31 (21.2)	40 (32.5)	0.04^b^
Eye problems, n (%)	8 (5.9)	5 (4.1)	0.59

Note: ^b^ further analysis on the exposed to pesticides group categories (mosquito only, non-mosquito, combined groups) adjusted for SES, mother’s age at pregnancy, breastfed for 6 months, house hold SHS exposure, water sources, fuel sources, garbage burning, and usage of open vehicle

## Discussion

This study shows that infants born from mothers exposed to household pesticides during pregnancy had lower head circumference at birth compare to those of non-exposed mothers, particularly with exposure to non-mosquito pesticides. Birth weight of infants born from mothers exposed to household pesticides was lower, but not statistically significantly, nor were there associations between exposure and aspects of growth, including weight gain, length gain, HC increment and weight-to-length gain rates.

The prospective cohort design is one of the strengths of this study. To our knowledge, not many studies have explored the effects of exposure to household pesticides during pregnancy on infant health, especially on birth size and infant growth [17, 25–27]. As most Indonesians use household pesticides to eradicate mosquitos or other insects [20], that population is relevant for studying the effects of antenatal exposure to household pesticides on infant health.

Like many other studies, exposure to household pesticides in this study was obtained from self-reported questionnaire [17, 26, 27]. We obtained questionnaire data at the 3rd trimester of pregnancy but none of the participants or the research staffs were aware of the study hypothesis; thus minimizing the possibility of information bias. On the final analysis, we adjusted for several potential confounders that were a priori defined including other potential EDCs, and the most important confounders, active and passive smoking during pregnancy.

There are several limitations to our study. Current household pesticides marketed in Indonesia contain pyrethroids (mostly), propoxur, carbamate, or in combination as active compounds, which are classified as non-persistent non-organophosphate pesticides. These non-persistent pesticides or their metabolites are difficult to measure, because they easily degrade with short half-lives, and only repeat (bio) sampling designs could characterize exposure in a more refined way [17, 26, 27]. In order to quantify the antenatal exposure, some studies recommended to measure the concentrations of pyrethroids cis-and trans-permethrin, piperonyl butoxide (PBO), propoxur, bendiocarb, and carbofuran in personal air sample, maternal saliva and urine during pregnancy or measure trans-permethrin concentration from umbilical cord [17, 26, 28, 29], but we did not performed any of these measurement. Repeated self-reported questionnaires throughout pregnancy with detailed information on the type, frequency, and amount of pesticides usage are considered preferable over a single entry questionnaire [17, 26, 28, 29]. Although further information on the type or brand or the amount of the pesticides used was unavailable, our findings provide essential first local evidence on the effect of early exposure of household pesticides on birth sizes and infant growth rate. Since we did not collect the information on the possibility of illegal use of organophosphate pesticides for household purposes, our study might also encounter general underestimation on the illegal use of organophosphate pesticides, which might have occurred on an individual basis. The possibility of other sources of exposure (e.g. occupational or agricultural) could also not entirely excluded, even though we assume that the likelihood was low given that most of our study population was unemployed and resided in the central of the Jakarta metropolitan area.

Many subjects from BRAVO had to be excluded due to incomplete data on exposure or outcomes, but that likely occurred at random with respect to this particular research question (Supplementay file 1: Table S1). Differential loss to follow-up [30] should be considered, but since the subjects and staff were unaware of the research question, loss to follow up likely occurred at random and was equally distributed between the exposed and non-exposed. Our study may have lacked robustness to explore possibly subtle effects on any infant growth parameters in the first 6 month of life, but we infer that there are no strong effects on infant growth parameters. Larger studies with sequential questionnaires during pregnancy and postnatal life are needed to explore further any antenatal effect of exposure to household pesticides on infant growth and health.

Epidemiological data on the association between exposure to household non-organophosphate pesticides and infant birth sizes and early growth are rare. Recent animal data suggest that exposure to pesticides during pregnancy and early life may impair growth and neurodevelopment in the offspring [14, 15, 17, 25, 29].

Antenatal exposure to organophosphate pesticides has been linked to lower HC at birth [14, 27] and neurodevelopmental disorder in infants [15], while antenatal exposure to permethrin (pyrethroid pesticides) in personal air and/or plasma was not associated with performance scores for the Bayley Mental Developmental Index (MDI) or the Psychomotor Developmental Index [17]. Higher exposure to piperonyl butoxide was linked with lower MDI scores compared with lower exposure [17]. HC correlates with brain weight, and brain size and head circumference are predictive of IQ and cognitive ability [31]. Our study shows a statistically significantly lower HC in infants from mothers exposed to house hold pesticides, with more pronounced effects found in those exposed to non-mosquito pesticides. The difference of HC at birth with exposure to non-mosquito pesticides is substantial and warrants further study.

Maternal exposure to combinations of modern, non-persistent pesticides during early pregnancy has been associated with lower birth weight, but increased body fat accumulation from birth to school age [25]. Higher levels of maternal exposure in early pregnancy were associated with lower child weight at birth [25]. Another study found that the use of pyrethroid products during pregnancy appears to be relatively safe because there were no increased rates of major malformations and no difference in birth weight and gestational age between the exposed and non exposed [32]. Our current study could not detect clear associations with birth length. This agrees with the result of a previous recent systematic review [31].

The mechanisms of how antenatal exposure to non-organophosphate pesticides may affect the fetal and infant growth remain speculative. There may be direct neurotoxic effects on to fetal brain development, disruption in the hypothalamic-pituitary-thyroid axis, on nuclear thyroid receptors as thyroid hormones are known to have major roles in brain development, and epigenetic influences on the fetal-maternal circulation [17, 31, 32]. We do note that exposure to non-organophosphate household pesticides seem to, if anything, affect fetal rather than infant development.

Our study also showed that infants of exposed mothers had higher frequencies of skin problems. We could not discern if that was due to antenatal or postnatal exposure to household pesticides, since we did not collect data on the postnatal exposure.

## Conclusions

In summary, our findings in a highly exposed population of Indonesian pregnant women suggest that antenatal exposure to household approved non-organophosphate pesticides could lead to lower head circumference at birth and life-long health implications. As household use of pesticides is particularly common in (sub) tropical low and middle income countries like Indonesia, also during pregnancy, our findings pertain to an important public health issue. Any toxic effects, even if subtle, on the developing fetus may have life-long health implications. Therefore, our findings need further in-depth investigation.

## Supplementary information

**Additional file 1: Table S1.** Baseline characteristics of all BRAVO participants.

## Data Availability

The datasets generated and/or analysed during the current study are not publicly available because the datasets contain multiple sensitive identifiers, but the datasets are available from the corresponding author on reasonable request.

## Abbreviations

EDC: Endocrine Disrupting Chemicals
HC: Head circumference
BRAVO: The Breastfeeding Attitude and Volume Optimization study
WLG: Weight gain rate adjusted for length gain rate
