# Supplementary material for: The effect of non-organophosphate household pesticides exposure during pregnancy on infants birth sizes and growth rate: a cohort study
Source: BMC Pregnancy Childbirth. 2020 Aug 20;20:476. doi: 10.1186/s12884-020-03162-w (PMC7441723; doi:10.1186/s12884-020-03162-w)
Supplement: Supplementary file 1 — Additional file 1: Table S1. Baseline characteristics of all BRAVO participants. [file 12884_2020_3162_MOESM1_ESM.docx]

**Table S1. Baseline characteristics of all BRAVO participants**

|  | |  | BRAVO participants | |  |
| --- | --- | --- | --- | --- | --- |
| Variable | |  | Included in household pesticides study  (n= 284) | Excluded in household pesticides study  (n=434) | p-value |
| MOTHER | | | | | |
| Maternal age (years) |  | | 28.7 (5.4) | 28.1 (5.7) | 0.22 |
| Parity^a^ |  | | 2 (2) | 2 (2) | 0.28 |
| History of abortion, n (%) |  | | 34 (11.9) | 48 (11.1) | 0.27 |
| BMI mother before pregnancy (kg/m^2^) | | | 22.5 (4.5) | 22.3 (5.4) | 0.80 |
| Family income (USD/month), n (%) | >223^b^ | | 41 (14.5) | 61 (14.1) | 0.33 |
| Educational level mother, n (%) | University level  Senior high school | | 21 (7.4)  188 (66.1) | 49 (11.0)  261 (62.6) | 0.05 |
| Passive smoking during pregnancy, n (%) | | | 210 (73.9) | 310 (71.4) | 0.35 |
| Actively smoking during pregnancy, n (%) | | | 5 (1.8) | 6 (1.4) | 0.36 |
|  |  | |  |  |  |
| INFANT |  | |  |  |  |
| Birth weight (grams) |  | | 3063 (451) | 3112 (432) | 0.57 |
| Birth length (cm) |  | | 48.3 (1.9) | 48.4 (2.1) | 0.30 |
| Head circumference (cm) |  | | 34.2 (1.8) | 33.9 (2.1) | 0.13 |
| Gestational age (weeks) |  | | 38.9 (1.4) | 38.8 (1.2) | 2. 0.65 |

Note: numbers are mean (SD) otherwise indicated, ^a^: median (interquartile range), ^b^family income 223 USD/month is the Indonesian minimum monthly wedges per capit
